# Immune checkpoint blockade and biomarkers of clinical response in non–small cell lung cancer

**DOI:** 10.1111/sji.12980

**Published:** 2020-10-24

**Authors:** Andreas Hallqvist, Anna Rohlin, Sukanya Raghavan

**Affiliations:** ^1^ Department of Oncology Institute of Clinical Sciences Sahlgrenska Academy University of Gothenburg Gothenburg Sweden; ^2^ Department of Oncology Sahlgrenska University Hospital Gothenburg Sweden; ^3^ Laboratory Medicine Institute of Biomedicine at the Sahlgrenska Academy University of Gothenburg Gothenburg Sweden; ^4^ Unit of Genetic Analysis and Bioinformatics Department of Clinical Genetics and Genomics Sahlgrenska University Hospital Gothenburg Sweden; ^5^ Department of Microbiology and Immunology Institute of Biomedicine at the Sahlgrenska Academy University of Gothenburg Gothenburg Sweden

**Keywords:** biomarkers, CD8 + T cells, immune therapy, non–small‐cell lung cancer, tumour mutation burden

## Abstract

Immunotherapy with PD‐1 and PD‐L1 inhibitors has revolutionized the treatment for patients with NSCLC the last years with increased overall survival and in particular increased number of long‐time survivors in patients with metastatic disease. It is now a treatment of choice for patients with distant metastases (stage IV) and in conjunction with chemoradiotherapy for patients with limited spread confined to the chest (stage III). PD‐1 inhibition has been proven to be superior to standard chemotherapy, both as a single treatment and when combined with either chemotherapy or CTLA‐4 inhibition. Despite the success of immunotherapy, the majority of patients do not respond or relapse within a short time frame. Biomarkers that would help to properly select patients with a high likelihood of clinical response to PD‐1 and PD‐L1 inhibitors are scarce and far from optimal, and only one (PD‐L1 expression) has reached clinical practice. Thus for immunotherapy to be effective, the discovery and validation of additional biomarkers is critical for patient selection and prediction of clinical response. In this mini‐review, we give an overview of current clinical management of NSCLC including treatment landscape with regard to immunotherapy, as well as discuss the current genetic and immune cell biomarker studies and their potential for introduction into clinical practice.

## 1 INTRODUCTION

In the last 5 years, the treatment options for NSCLC patients have increased remarkably after the introduction of immune therapy ('immune checkpoint blockade—ICB'). Although the indications where ICB is recommended are expanding every year, a majority of NSCLC patients do not respond to ICB. Biomarkers for patient selection and prediction of clinical response will be important to tailor treatment schedules for individual patients. This mini‐review aims to give an overview of ICB in NSCLC approved by the European Medical Agency (EMA) and discuss potential future biomarkers.

Lung cancer is still the malignancy with the highest mortality rate in all of the Nordic countries, resulting in >12 000 deaths annually.^1^ The incidence show a similar pattern in the last decade with rising figures, but the number of new cases seems to have reached a plateau. NSCLC predominantly encompassing adenocarcinomas and squamous cell carcinomas (SCC) accounts for 80%‐85% of all lung cancer cases. An estimated 60% of the patients with NSCLC have distant metastases by the time of diagnosis (stage IV), 25% have advanced growth of the primary tumour and/or advanced regional lymph node metastases (stage III—locally advanced), and the remaining 15% have tumours confined to the lung or with limited regional nodal spread (stages I‐II).^2^ As the majority of patients unfortunately are metastatic upon diagnosis, the cure rates are low and all stages are at a high risk of relapse and progression despite modern therapy.

## 2 OVERVIEW OF TREATMENT FOR NSCLC

In principle, stage I‐III patients are considered to have a curative potential. For early (stages I‐II) NSCLC, surgery is the current standard procedure. An alternative, mostly used in medically inoperable patients, for stage I‐IIa tumours is stereotactic body radiotherapy (SBRT). This is a precise delivery of radiation to very high doses in a short period of time and has showed excellent results in terms of local control with low toxicity. In surgically treated patients, it is standard procedure to give adjuvant chemotherapy postoperatively in patients with stages II‐IIIA. There are no data on adjuvant chemotherapy after SBRT, and chemotherapy is rarely given to these medically inoperable patients due to expected toxicities and comorbidities. There are no approved indications for immunotherapy in early‐stage NSCLC.

The standard treatment regimen for stage III diagnosed patients is combined chemoradiotherapy if they have an acceptable lung function and are fit enough to receive this rather intense schedule. The treatment strategies for stage III patients have varied somewhat during the last decade, and the best approach is currently unknown but there are some established protocols: the chemotherapy should be given with a platinum backbone doublet, and preferably concurrently with radiotherapy to a total dose of 60‐68 Gy. The most recent development in stage III disease is the incorporation of immunotherapy with the approval of the PD‐L1 inhibitor durvalumab, which improves survival if added after the concurrent schedule of chemoradiotherapy.^3^

The former standard treatment in stage IV disease without specific targetable oncogenic drivers (eg, EGFR mutations or ALK fusions) was combinations of platinum‐based chemotherapy. The treatment strategy has however changed considerably the last years following the introduction of immunotherapy due to the impressive clinical results with improved overall survival both in the first‐ and in the second‐line setting. In short, the present recommendation for all stage IV diagnosed patients without specific oncogenic drivers is that they are assessed for start of immunotherapy as part of their first‐line treatment.

## 3 ICB FOR THE TREATMENT OF NSCLC

### 3.1 PD‐1 and PD‐L1

Following the successful clinical trials in the treatment of malignant melanoma, introducing ICB as a new class of pharmaceuticals,^4^, ^5^ it was thereafter established as a treatment for NSCLC patients. The first positive trials and associated approved indication were in the second‐line setting where the PD‐1 inhibitors nivolumab and pembrolizumab and later the PD‐L1 inhibitor atezolizumab all showed significantly improved overall survival in stage IV disease compared with chemotherapy (monotherapy) with docetaxel. In addition, the now well‐known elevated and plateaued tail of the survival curve indicated a consistent subgroup of long‐time survivors.^6^, ^7^, ^8^, ^9^ In contrast, another PD‐L1 inhibitor avelumab did not meet the primary endpoint in a corresponding trial.^10^ Yet in 2018, immunotherapy moved forward as a treatment of choice when pembrolizumab proved to be superior to doublet chemotherapy in the first‐line setting in stage IV NSCLC patients with a high expression of PD‐L1 (≥50%).^11^ Pembrolizumab has since then been the only approved monotherapy ICB for first‐line treatment as nivolumab and durvalumab have failed to improve survival in a similar setting.^12^, ^13^ However, recently monotherapy with atezolizumab was found to improve overall survival in patients with high PD‐L1 expression compared to standard doublet chemotherapy^14^ and will likely be an approved alternative for first‐line treatment of stage IV NSCLC patients in the near future. To improve efficacy of treatments for the patients, combinations of ICB with chemotherapy have been assessed. Triplet regimens with platinum doublets and pembrolizumab or atezolizumab, as well as quadruplet therapy including the anti‐VEGF antibody bevacizumab, proved to be successful with improved overall survival over chemotherapy alone.^15^, ^16^, ^17^, ^18^ Chemotherapy and immunotherapy (PD‐1 or PD‐L1) combinations are now considered standard therapy for fit stage IV patients without contraindications or oncogenic drivers, where pembrolizumab as monotherapy stays as an alternative for patients with high PD‐L1 expression. To date, the only indication for immunotherapy in stage III disease is adjuvant durvalumab after combined chemoradiotherapy.^3^

### 3.2 CTLA‐4

There are currently no approved CTLA‐4–directed therapies in lung cancer, but the combination of ipilimumab (CTLA‐4 inhibitor) and nivolumab is under evaluation by the EMA, as a survival benefit has been observed in stage IV NSCLC compared with standard chemotherapy.^19^, ^20^ However, whether the combination of ipilimumab and nivolumab would be superior to the current standard of triple combination with chemotherapy and PD‐1 or PD‐L1 inhibition is unknown and remains to be investigated. Another CTLA‐4 inhibitor tremelimumab was assessed in combination with durvalumab in stage IV NSCLC but failed to significantly improve overall survival compared with chemotherapy albeit numerically increased long‐time survival.^13^ Although there is a potential for CTLA‐4 blockade in combination with PD‐1 or PD‐L1 inhibitors in the treatment of NSCLC, it is currently unclear how patients can be selected and how it would be compared with the existing therapies.

## 4 BIOMARKERS OF CLINICAL RESPONSE TO ICB IN NSCLC

In NSCLC, the only predictive biomarker in clinical routine practice is PD‐L1 expression where expression on tumour cells and sometimes including immune cells is quantified to obtain a percentage of PD‐L1 positivity that is used to make treatment decisions. Several studies have shown that increasing PD‐L1 expression is predictive of longer progression‐free survival (PFS) and overall survival (OS) after PD‐1 inhibition when compared to chemotherapy in stage IV NSCLC patients, and a cut‐off of 50% is used to select patients for first‐line monotherapy with pembrolizumab.^11^ However, PD‐L1 expression is far from an optimal biomarker for patient selection since patients negative for PD‐L1 can also present durable response,^6^ and for the combination therapies, assessment of PD‐L1 expression is of negligible value.^16^, ^17^ In addition, varying definitions for positivity of PD‐L1 staining using percentage of PD‐L1–positive tumour cells (TPS—tumour proportion score) or positive tumour and infiltrating immune cells (CPS—combined positive score), different testing platforms and antibodies also make it difficult to compare between various clinical trials.^21^ The clinical implication of the different PD‐L1 thresholds and techniques varies among countries, clinics and even among clinicians, ranging from a pragmatic standpoint to a more strict position requiring certified and specific antibodies for each drug. Considering the insufficiency of PD‐L1 expression, other biomarkers are urgently needed, and here, we discuss recent studies and elaborate on some promising candidates for NSCLC patients.

## 5 GENETIC BIOMARKERS OF RESPONSE TO ICB

Broad genomic sequencing approaches including whole‐genome sequencing (WGS), whole‐exome sequencing (WES) and sequencing of smaller gene panels to identify DNA sequence changes in the tumour have been investigated. Information regarding the genetic landscape can be used in a broader sense to identify common or specific patterns in the tumour for each patient or to identify gene‐specific mutations (DNA sequence variants predicted to be pathogenic). These DNA sequence changes in individual tumours can be used as biomarkers of clinical response to ICB. The current genetic biomarkers under investigation in NSCLC patients after ICB are outlined below, where analysis of the tumour mutational burden (TMB) is the second most investigated biomarker after PD‐L1 expression.

### 5.1 Tumour mutational burden

The number of DNA variants (DNA sequence changes) in the tumour can be calculated and is often referred to as the TMB^22^. The DNA variants calculated is often the non‐synonymous variants that can be found in the coding gene regions of the sequenced tumour DNA. A high number of DNA changes refer to a high TMB. In studies of lung cancer patients after ICB as a second‐line treatment, higher TMB was correlated with durable clinical benefit, with longer OS and a higher number of patients responding to the treatment.^22^, ^23^, ^24^ To date, >20 clinical studies have been performed, and in a recent randomized clinical trial (nivolumab and ipilimumab in combination) in the first‐line setting, a threshold of ≥10 mutations/Mb was predictive of longer PFS in NSCLC patients.^25^ Tumours with a high TMB presumably benefit from ICB therapy since it is correlated with the presence of a high number of neoantigens that could be recognized by CD8^+^ T cells that favour tumour immunity. Any acquired somatic genetic DNA variant in the tumour cells can be a potential neoantigen, and a target for the tumour‐specific CD8^+^ T cells. However, only a small fraction of mutations results in neoantigens recognized by the T cells and influenced by several factors such as type of mutation, the clonal distribution of the mutation and the contribution of other antigens (non‐mutant) in the tumour microenvironment.^26^ Currently, the TMB assessment is not standardized across research and clinical studies and there are several factors that influence the measurement of the TMB analysis. The results from the analysis can therefore be very different even between analysis from the same patient sample and between samples. Factors that influence the results are sample quality and quantity, sequencing platform, genome coverage, bioinformatics analysis pipelines used and definitions of the threshold that determines the high or the low TMB.^27^ Nevertheless, the Food and Drug Administration (FDA) has recently approved TMB as an agnostic biomarker for selection of patients with a TMB cut‐off ≥10 mutations/Mb for treatment with pembrolizumab.^28^ The use of TMB to select patients likely to respond to ICB is a first positive step in the right direction for finding alternative biomarkers of clinical response, although currently it is still uncertain whether the strategy will withstand larger trials and receive approval by the EMA. We expect that TMB as a stand‐alone biomarker will probably be of modest clinical value and that most likely it would be in combination with analysis of other biomarkers for a better predictive value.

### 5.2 Germline genotype of HLA‐I

One of the established determinant of response to ICB therapy is the number of tumour‐derived neoantigens, derived from the somatic mutations of the tumour, which are then processed intracellularly for presentation on the major histocompatibility complex class I (MHC I).^24^ The human leucocyte antigen (HLA‐I) gene family is the human form of the major MHC I, binds specific peptides for presentation of intracellular tumour antigens to CD8 + cytotoxic T cells.^24^ The HLA‐I in humans is present on three different loci A, B and C that are highly polymorphic within the peptide‐binding domains. Interestingly, the heterozygosity of the HLA‐I genes has been shown to influence the survival and response to ICB presumably due to ability to present varied peptide antigens, including neoantigens from the tumour.^29^ Indeed, homozygosity at a HLA‐I locus in combination with low TMB was associated with decrease in survival after ICB compared with cohorts of patients that were heterozygous at any of HLA‐I locus and presented a high TMB.^29^ The effect of HLA‐I heterozygosity and high TMB was superior in predicting response to ICB compared with high TMB alone. Loss of heterozygosity in at least one HLA‐I locus (HLA‐A, HLA‐B or HLA‐C) was sufficient to reduce probability for survival after ICB.^29^ Thus, it is becoming apparent that in addition to the TMB status of the patients one may also need to analyse the HLA‐I genotype to offer ICB therapy to the patients most likely to respond.

### 5.3 Mutations in DNA mismatch repair and replication genes

Studies of additional clinically relevant genomic biomarkers are associated with mutations in genes affecting DNA repair and proofreading, which in general leads to a high TMB. Tumours with mutations in DNA polymerase E (*POLE*) or DNA polymerase D1 (*POLD1)* have a very high TMB, and a hypermutated phenotype has been associated with a favourable outcome after ICB therapy.^30^, ^31^, ^32^ At least 6% NSCLC patients harbour *POLE* and *POLD1* mutations.^33^ Mutations in *POLE* and *POLD1* are potential predictive biomarkers for positive outcome to ICB therapy.

### 5.4 Additional clinically relevant genomic alterations

Favourable clinical benefit of PD‐1/PD‐L1 inhibition has been observed in NSCLC patients with *TP53* mutations or combinations of *KRAS* and *TP53* mutations in the same tumour independent of TMB.^34^, ^35^, ^36^ In contrast, co‐occurrence of mutations in *KRAS* and *STK11* is associated with lack of response to ICB. Thus, tumours with *STK11* mutations seem to be associated with a worse outcome of ICB therapy and it has also been shown that co‐occurrence of *STK11* with, for example, *KEAP1* or *KRAS* may be prognostic rather than predictive.^37^, ^38^ Further studies on the association of *STK11* mutations in response to ICB are warranted since other mutations in the tumour will also influence the clinical response.^39^, ^40^ The clinically relevant *EGFR* mutations have also been shown to correlate with a worse clinical response for patients to ICB, whereas certain *BRAF* and *MET* mutations are associated with better response, regardless of TMB status.^41^ The implication so far is that NSCLC with oncogenic drivers such as *EGFR* mutations should not be treated with monotherapy ICB, but perhaps combined therapies with, for example, chemotherapy or other immune checkpoint inhibitors may have a promising future role.

### 5.5 Liquid biopsies

Liquid biopsy is a non‐invasive method for analysis of biomarkers of response to ICB in the circulation of patients. The two most well‐developed methods utilizing liquid biopsy from patients are the isolation of circulating tumour cells (CTCs) and circulating cell‐free tumour DNA (ctDNA). CtDNA is shed by tumour cells and can be isolated from the blood of patients during therapy^42^. Due to the simplicity of a blood draw, it can be repeated as needed to follow the tumour response to PD‐1/PD‐L1 inhibition. Studies to date have investigated the ctDNA amount, the variant allele frequencies of specific mutations identified in the tumour in individual patients, or screened for mutation using large gene panels. In NSCLC patients, ctDNA amounts in the plasma have often been measured at baseline and at one to several time points during treatment. The results imply that a high or increased concentration of ctDNA at follow‐up (in general around 2 month) compared with baseline is associated with poor response and no long‐term clinical benefit in contrast to responders where a decrease in ctDNA or undetectable amounts were found.^43^, ^44^, ^45^, ^46^ CtDNA analysis could be a useful biomarker to predict early clinical response, but further studies with larger populations and longer follow‐up are needed. There are still technical challenges and a need for standardization of the different ctDNA detection techniques. The TMB can also be analysed in ctDNA. TMB calculation from ctDNA, derived from blood specimens (bTMB), has recently been evaluated in NSCLC patients after ICB. These analyses from several ongoing trials show comparable results with TMB calculation from tumour samples, with bTMB >16 mut/Mb as a cut‐off to select responders to atezolizumab.^47^, ^48^ If the predictive value could be proven, then bTMB would be useful to dynamically follow the clinical response to treatment.

Circulating tumour cells (CTCs) are rare cells present in very low concentration in the bloodstream. CTC has mainly been isolated for the analysis of PD‐L1 expression in relation to clinical outcome after ICB. Higher expression of PD‐L1 on CTCs at 6 months post‐treatment was associated with worse prognosis, while a distinction could not be made at an earlier time point, which was 3 months post‐treatment compared with pretreatment sampling.^49^, ^50^ The detection of CTCs in circulation is technically challenging, and standardization of methods between laboratories around the world is essential to be able to introduce the analysis into routine clinical practice. Since many different methods of purifications of CTCs exist, a discrepancy in the enrichment of CTCs may also effect the PD‐L1 assessment.^51^

### 5.6 Immune gene signatures

In Table 1, we summarize the exhaustive data available through databanks analysing immune gene signatures at the DNA or RNA level. A correlation with pre‐existing defined signatures has been observed in several studies, and in addition, correlation with expression of specific genes has also been found, even though the clinical impact remains to be seen (Table 1). The smoke signature associated with high TMB and high neoantigen load has been associated with response to ICB.^24^

**Table 1:** Immune gene signatures in the tumour as biomarker of clinical response to ICB in NSCLC

Material and method	Sample size	Main findings	ICB	Reference
Tumour DNA				
Genomic dataset	113 (TCGA)	Combined TMB and APOBEC (family of cytidine deaminases) signature was associated with longer PFS. Mutations in IFNGR1 or VTCN1 (B7‐H4) only found in responders, PTEN mutations only found in non‐responders	CTLA‐4, PD‐1 (not specified)	68
Whole‐exome sequencing (WES)	77	Combination of high TMB, a high number of neoantigens, mutational signatures 1A and 1B (COSMIC signatures), mutations in DNA repair pathways and a low number of TCR clones correlates with longer OS and PFS	Pembrolizumab or with nivolumab and ipilimumab	69
Tumour RNA				
RNA sequencing (whole transcriptome)	97	4‐gene IFN‐γ signature correlated with longer OS and PFS compared to patients with IFN‐γ signature profile.	Durvalumab	70
Predesigned RNA panel (Nanostring 730‐immune panel)	65	23 immune‐related genes or signatures linked to PFS including PD1, or signatures, mostly targeting CD8 and CD4 T cells, and IFN activation was independent of cancer type.	Pembrolizumab or nivolumab	71
Quantitative PCR	17	IFNG mRNA expression emerged as the only biomarker that significantly influenced treatment outcome in NSCLC. PFS was significantly longer in patients with high versus low IFNG expression.	Nivolumab	72
RNA Sequencing (whole transcriptome)	113 (TCGA)	CD8 + T cells, CD4 memory‐activated T cells, NK cells and M1 macrophages enriched in the patients with high mutational APOBEC activity, while CD4 resting memory T cells, monocytes and regulatory T cells enriched in the patients with low APOBEC mutational activity.	CTLA‐4, PD‐1 (not specified)	68
RNA Sequencing (whole transcriptome)	34 (cbio portal)	APOBEC3B and APOBEC mutational signature enriched in patients with durable clinical benefit (DCB) compared with patients with no durable benefit (NCB).	PD‐1 (not specified)	73
RNA panel sequencing	220 (including NSCLC)	The T cell–inflamed gene expression profile containing IFN‐γ–responsive genes common in tumours responsive to ICB.	Pembrolizumab	74
RNA panel sequencing	21	Longer PFS in patients with high M1 macrophage and peripheral T cell signature. CD137 and PSMB9 gene expression higher in responders than in non‐responders.	Pembrolizumab	75

Abbreviations: ICB, immune checkpoint blockade; NSCLC, non–small‐cell lung cancer; PFS, progression‐free survival; TMB, tumour mutational burden.

## 6 IMMUNE CELL BIOMARKERS OF RESPONSE TO ICB

The difficulty in obtaining biopsies post‐treatment particularly from lung cancer patients during ICB underscores the importance of characterizing immune cells in the blood as a means for potential early assessment of clinical response. The frequent sampling will also reflect dynamic changes over time, allowing identification of certain immune populations correlated with tumour shrinkage. Analyses of immune cell subsets in circulation before and after treatment in individual patients and the presence of soluble mediators of inflammation in the plasma are currently under investigation.

### 6.1 Neutrophils

In NSCLC patients, neutrophils in the tumour tissue have been found to inversely correlate with the frequency of T cells, indicating that neutrophils might be a suppressive factor for lymphocytes infiltrating the tumour tissue.^52^ In a preclinical lung tumour model, high neutrophil infiltration in the lungs was associated with resistance to PD‐1 blockade.^53^ Similar analysis of neutrophils in the blood has been suggested as a marker for inflammation and investigated for its usefulness as a biomarker of clinical response to ICB. A high pretreatment neutrophil‐to‐lymphocyte ratio (NLR) was shown to correlate with poor overall survival in NSCLC patients with metastatic or non‐resectable tumour treated with nivolumab.^54^, ^55^ Thus, analysis of circulating neutrophils could be a potential biomarker for patient selection for ICB. However, it is most likely that high NLR ratio by itself cannot predict response to PD‐1/PD‐L1 inhibition and would need to be combined with analysis of additional specific immune cell subsets as discussed below.

### 6.2 T cells

Data from clinical trials suggest that patients undergoing PD‐1/PD‐L1 inhibition share some common features of the immune response that is independent of the tumour type. In melanoma and lung cancer patients, clinical benefit after PD‐1 blockade was associated with proliferation/expansion of PD‐1^+^CD8^+^ T cells in the blood.^56^, ^57^ In contrast, the majority of the patients with no clinical benefit had delayed or absent proliferating PD‐1^+^CD8^+^ T cells. CD8^+^ T cells are dependent on CD4^+^ T cells for providing help through cell‐cell interaction and cytokine secretion for activation and differentiation. In this regard, interestingly, the long‐term survival after nivolumab treatment (>500 days) was correlated with higher frequencies of pretreatment CD4^+^CD62L^low^ cells (activated memory cells) compared with short‐term response (<500 days),^58^ indicating that activation of both CD8^+^ and CD4^+^ T cells is important for tumour immunity after ICB.

In the tumour microenvironment, the antigen‐specific effector T cells are suppressed either due to the PD‐1‐PD‐L1 interactions or due to immune suppressive cell populations. CD4^+^PD‐1^hi^ T cells with possible immune suppressive effects on CD8^+^ T cells have been shown to accumulate in the lung as a function of tumour burden, and inhibit effector T cell functions.^59^ Treatment with nivolumab or pembrolizumab reduced the frequency of CD4^+^PD‐1^hi^ cells in the blood and tumour tissue, but the effects on OS and PFS were not reported.^59^ In another study, high frequency of CD8^+^PD‐1^hi^ cells in pretreatment biopsies from stage IV NSCLC patients undergoing ICB predicted response to therapy and correlated with increased overall survival and durable responses.^60^ The CD8^+^PD‐1^hi^ T cells located in the tumour tissue displayed a significantly higher clonality, with the top 30 clones contributing close to 90% of the entire T cell receptor repertoire compared with CD8^+^PD‐1^neg^ T cells. The CD8^+^PD‐1^hi^ T cells in the tumour of NSCLC patients thus might represent the tumour antigen–specific CD8^+^ T cells released from the immune inhibitory effects of PD‐1‐PD‐L1 interaction.

Analysis of antigen specificity of tumour‐infiltrating CD8^+^ cells in NSCLC is not only important but also technically challenging. By combining sequencing to predict peptide binding to host HLA with tetramer staining and mass cytometry, Fehlings et al in a unique study characterized the neoantigen‐specific T cells in circulation after PD‐L1 inhibition.^61^ Interestingly, the tumour antigen–specific CD8^+^ T cells identified by tetramer staining in responder patients were often of a differentiated effector cell phenotype compared with antigen‐specific CD8^+^ T cells from patients with progressive disease that displayed a more memory‐like phenotype.^61^

To date, studies reporting changes in immune cell subsets after PD‐1 inhibition in NSCLC patients have mostly been in discovery cohorts. We can expect that as data start to emerge from phase 2/3 clinical trials, there will be an effort for further validation of the early and most promising immune cell biomarkers of clinical response, particularly those related to T cell function.

### 6.3 Soluble proteins detected in the plasma

The technically less challenging analysis of soluble biomarkers of immune responses after ICB in the plasma/serum compared with flow cytometry makes it attractive to explore in the context of ICB. Soluble PD‐L1 (sPD‐L1) or expression on extracellular vesicles, soluble granzyme B (sGranzyme B) and circulating cytokines and chemokines have been analysed and shown to be promising as biomarkers of clinical response after ICB as discussed below.

#### 6.3.1 sPD‐L1

Pretreatment low plasma sPD‐L1 concentrations in advanced/stage IV NSCLC patients treated with nivolumab correlated with longer overall survival and higher overall response rate and associated with patients achieving complete response, partial response or stable disease compared with patients presenting with progressive disease.^62^ Thus, measurement of sPD‐L1 could bring value as biomarker in patients receiving ICB.

#### 6.3.2 sGranzyme B

In the plasma of NSCLC patients treated with nivolumab, sGranzyme B was found to be modestly higher in responders than in non‐responders.^62^ However, increasing concentrations of sGranzyme B in the circulation during nivolumab treatment was unexpectedly correlated with shorter progression‐free survival and overall survival compared to patients with decreasing concentrations of sGranzyme B during treatment.^62^ This dichotomy in the response pre‐ and post‐treatment could possibly indicate lack of utilization of the Granzyme B, the effector molecule necessary for tumour killing specifically in the tumour leading to poor overall survival.

#### 6.3.3 Cytokines and chemokines

There are little data available on levels of soluble cytokines and chemokines as biomarkers of clinical response to ICB in NSCLC patients although it has been analysed in relation to immune‐related adverse events in patients receiving a combination of PD‐1 and CTLA‐4 inhibition. A higher IL‐6 and IL‐12 levels in serum is associated with worse survival independent of therapy.^63^ Recently, studies have also shown that extracellular vesicles or exosomes derived from the tumour can express PD‐L1 and to the same extent as the tumour tissue.^64^ Further, two preliminary studies report that a decrease in exosomal PD‐L1 expression was associated with partial response after ICB.^65^, ^66^ In NSCLC patients treated with nivolumab or pembrolizumab, responders had significantly lower levels of IL‐8 between baseline and the first tumour evaluation, while non‐responders had significantly high levels of IL‐8. Furthermore, an early lowering of IL‐8 in circulation was associated with longer OS compared to patients with increasing levels of IL‐8.^67^ The function of IL‐8 in the response to ICB is however currently unknown.

## 7 CHALLENGES AND FUTURE DIRECTION

Immune checkpoint blockade has proven to be very successful for the treatment of NSCLC patients, and their use is continuously expanding into new indications. In the future, immunotherapy will be included in the treatment of earlier stage of NSCLC, neoadjuvant and/or adjuvant after surgery and in further combination regimens including radiotherapy, where numerous trials are ongoing. However, despite the paradigm shift we have seen in the treatment of NSCLC, the majority of patients treated today will not respond to immunotherapy or relapse in a short time frame, and one of the biggest challenges is proper patient selection. We propose that a combined signature that may include PD‐L1 expression, TMB analysis, identification of specific mutations, immune cell populations in the blood and soluble protein markers in the plasma will in the future help guiding clinicians to select the appropriate treatment for the individual patient; a step towards precision medicine. The continuous research in the field of biomarkers is also highly relevant in addressing the vital question of individual patient's response and/or resistance to ICB.

## Data Availability

The corresponding author can be contacted for data availability.

## References

[1] Global cancer observatory . Available from https://gcoiarcfr/

[2] Swedish Lung cancer registry. Available from: http://www.cancercentrum.se/vast/cancerdiagnoser/lunga‐och‐lungsack/kvalitetsregister.

[3] Antonia SJ , Villegas A , Daniel D , et al. Overall survival with durvalumab after chemoradiotherapy in stage III NSCLC. N Engl J Med. 2018;13(379):2342‐2350. 10.1056/NEJMoa1809697 30280658

[4] Robert C , Long GV , Brady B , et al. Nivolumab in previously untreated melanoma without BRAF mutation. N Engl J Med. 2015;22(372):320‐330. 10.1056/NEJMoa1412082 25399552

[5] Robert C , Schachter J , Long GV , et al. Pembrolizumab versus Ipilimumab in advanced melanoma. N Engl J Med. 2015;25(372):2521‐2532. 10.1056/NEJMoa1503093 25891173

[6] Rittmeyer A , Barlesi F , Waterkamp D , et al. Atezolizumab versus docetaxel in patients with previously treated non‐small‐cell lung cancer (OAK): a phase 3, open‐label, multicentre randomised controlled trial. Lancet. 2017;21(389):255‐265. 10.1016/S0140-6736(16)32517-X PMC6886121 27979383

[7] Borghaei H , Paz‐Ares L , Horn L , et al. Nivolumab versus docetaxel in advanced nonsquamous non‐small‐cell lung cancer. N Engl J Med. 2015;22(373):1627‐1639. 10.1056/NEJMoa1507643 PMC5705936 26412456

[8] Brahmer J , Reckamp KL , Baas P , et al. Nivolumab versus docetaxel in advanced squamous‐cell non‐small‐cell lung cancer. N Engl J Med. 2015;9(373):123‐135. 10.1056/NEJMoa1504627 PMC4681400 26028407

[9] Herbst RS , Baas P , Kim DW , et al. Pembrolizumab versus docetaxel for previously treated, PD‐L1‐positive, advanced non‐small‐cell lung cancer (KEYNOTE‐010): a randomised controlled trial. Lancet. 2015;387(10027):1540‐1550. 26712084 10.1016/S0140-6736(15)01281-7

[10] Barlesi F , Vansteenkiste J , Spigel D , et al. Avelumab versus docetaxel in patients with platinum‐treated advanced non‐small‐cell lung cancer (JAVELIN Lung 200): an open‐label, randomised, phase 3 study. Lancet Oncol. 2018;19:1468‐1479. 30262187 10.1016/S1470-2045(18)30673-9

[11] Reck M , Rodriguez‐Abreu D , Robinson AG , et al. Pembrolizumab versus chemotherapy for PD‐L1‐positive non‐small‐cell lung cancer. N Engl J Med. 2016;10(375):1823‐1833. 10.1056/NEJMoa1606774 27718847

[12] Carbone DP , Reck M , Paz‐Ares L , et al. First‐Line nivolumab in stage IV or recurrent non‐small‐cell lung cancer. N Engl J Med. 2017;22(376):2415‐2426. 10.1056/NEJMoa1613493 PMC6487310 28636851

[13] Rizvi NA , Cho BC , Reinmuth N , et al. Durvalumab with or without tremelimumab vs standard chemotherapy in first‐line treatment of metastatic non‐small cell lung cancer: the MYSTIC phase 3 randomized clinical trial. JAMA Oncol. 2020;6(5):661‐674. 32271377 10.1001/jamaoncol.2020.0237 PMC7146551

[14] Spigel D , de Marinis F , Giaccone G , et al. ImPOWER110: interim overall survival (OS) analysis of a phase III study of atezolizumab (atezo) vs platinum‐based chemotherapy (chemo) as first‐line (1L) treatment (tx) in PDL1‐selected NSCLC. Ann Oncol. 2019;30(suppl_5):v851‐v934.

[15] Gandhi L , Rodriguez‐Abreu D , Gadgeel S , et al. Pembrolizumab plus chemotherapy in metastatic non‐small‐cell lung cancer. N Engl J Med. 2018;31(378):2078‐2092. 10.1056/NEJMoa1801005 29658856

[16] Paz‐Ares L , Luft A , Vicente D , et al. Pembrolizumab plus chemotherapy for squamous non‐small‐cell lung cancer. N Engl J Med. 2018;22(379):2040‐2051. 10.1056/NEJMoa1810865 30280635

[17] Socinski MA , Jotte RM , Cappuzzo F , et al. Atezolizumab for first‐line treatment of metastatic nonsquamous NSCLC. N Engl J Med. 2018;14(378):2288‐2301. 10.1056/NEJMoa1716948 29863955

[18] West H , McCleod M , Hussein M , et al. Atezolizumab in combination with carboplatin plus nab‐paclitaxel chemotherapy compared with chemotherapy alone as first‐line treatment for metastatic non‐squamous non‐small‐cell lung cancer (IMpower130): a multicentre, randomised, open‐label, phase 3 trial. Lancet Oncol. 2019;20:924‐937. 31122901 10.1016/S1470-2045(19)30167-6

[19] Hellmann MD , Paz‐Ares L , Bernabe Caro R , et al. Nivolumab plus ipilimumab in advanced non‐small‐cell lung cancer. N Engl J Med. 2019;21(381):2020‐2031. 10.1056/NEJMoa1910231 31562796

[20] Reck M , Ciuleanu TE , Cobo Dols M , Schenker M , Zurawski B . Nivolumab (NIVO) + ipilimumab (IPI) + 2 cycles of platinum‐doublet chemotherapy (chemo) vs 4 cycles chemo as first‐line (1L) treatment (tx) for stage IV/recurrent non‐small cell lung cancer (NSCLC): CheckMate 9LA. J Clin Oncol. 2020;38(15):9501.

[21] Lantuejoul S , Sound‐Tsao M , Cooper WA , et al. PD‐L1 testing for lung cancer in 2019: perspective from the IASLC Pathology Committee. J Thorac Oncol. 2020;15:499‐519. 31870882 10.1016/j.jtho.2019.12.107

[22] Hellmann MD , Callahan MK , Awad MM , et al. Tumor mutational burden and efficacy of nivolumab monotherapy and in combination with ipilimumab in small‐cell lung cancer. Cancer Cell. 2018;14(33):853‐861.e4. 10.1016/j.ccell.2018.04.001 PMC6750707 29731394

[23] Hellmann MD , Nathanson T , Rizvi H , et al. Genomic features of response to combination immunotherapy in patients with advanced non‐small‐cell lung cancer. Cancer Cell. 2018;14(33):843‐852.e4. 10.1016/j.ccell.2018.03.018 PMC5953836 29657128

[24] Rizvi NA , Hellmann MD , Snyder A , et al. Cancer immunology. Mutational landscape determines sensitivity to PD‐1 blockade in non‐small cell lung cancer. Science. 2015;3(348):124‐128. 10.1126/science.aaa1348 PMC4993154 25765070

[25] Ready N , Hellmann MD , Awad MM , et al. First‐Line nivolumab plus ipilimumab in advanced non‐small‐cell lung cancer (CheckMate 568): outcomes by programmed death ligand 1 and tumor mutational burden as biomarkers. J Clin Oncol. 2019;20(37):992‐1000. 10.1200/JCO.18.01042 PMC6494267 30785829

[26] Schumacher TN , Scheper W , Kvistborg P . Cancer neoantigens. Annu Rev Immunol. 2019;26(37):173‐200. 10.1146/annurev-immunol-042617-053402 30550719

[27] Buttner R , Longshore JW , Lopez‐Rios F , et al. Implementing TMB measurement in clinical practice: considerations on assay requirements. ESMO Open. 2019;4:e000442. 30792906 10.1136/esmoopen-2018-000442 PMC6350758

[28] Marabelle A , Fakih M , Lopez J , et al. Association of tumour mutational burden with outcomes in patients with advanced solid tumours treated with pembrolizumab: prospective biomarker analysis of the multicohort, open‐label, phase 2 KEYNOTE‐158 study. Lancet Oncol. 2020;21(10):1353‐1365. 32919526 10.1016/S1470-2045(20)30445-9

[29] Chowell D , Krishna C , Pierini F , et al. Evolutionary divergence of HLA class I genotype impacts efficacy of cancer immunotherapy. Nat Med. 2019;25:1715‐1720. 31700181 10.1038/s41591-019-0639-4 PMC7938381

[30] Liu L , Ruiz J , O'Neill SS , et al. Favorable outcome of patients with lung adenocarcinoma harboring POLE mutations and expressing high PD‐L1. Mol Cancer. 2018;17:81. 29650000 10.1186/s12943-018-0832-y PMC5897927

[31] Song Z , Cheng G , Xu C , Wang W , Shao Y , Zhang Y . Clinicopathological characteristics of POLE mutation in patients with non‐small‐cell lung cancer. Lung Cancer. 2018;118:57‐61. 29572003 10.1016/j.lungcan.2018.02.004

[32] Yao J , Gong Y , Zhao W , et al. Comprehensive analysis of POLE and POLD1 gene variations identifies cancer patients potentially benefit from immunotherapy in Chinese population. Sci Rep. 2019;31(9):15767. 10.1038/s41598-019-52414-z PMC6823448 31673068

[33] Wang Feng , Zhao Qi , Wang Ying‐Nan , Jin Ying , He Ming‐Ming , Liu Ze‐Xian , Xu Rui‐Hua . Evaluation of POLE and POLD1 Mutations as Biomarkers for Immunotherapy Outcomes Across Multiple Cancer Types. JAMA Oncology. 2019;5 (10):1504. 10.1001/jamaoncol.2019.2963 PMC6696731 31415061

[34] Dong ZY , Zhong WZ , Zhang XC , et al. Potential predictive value of TP53 and KRAS mutation status for response to PD‐1 Blockade immunotherapy in lung adenocarcinoma. Clin Cancer Res. 2017;15(23):3012‐3024. 10.1158/1078-0432.CCR-16-2554 28039262

[35] Fang C , Zhang C , Zhao WQ , Hu WW , Wu J , Ji M . Co‐mutations of TP53 and KRAS serve as potential biomarkers for immune checkpoint blockade in squamous‐cell non‐small cell lung cancer: a case report. BMC Med Genomics. 2019;16(12):136. 10.1186/s12920-019-0592-6 PMC6794845 31619231

[36] Assoun S , Theou‐Anton N , Nguenang M , et al. Association of TP53 mutations with response and longer survival under immune checkpoint inhibitors in advanced non‐small‐cell lung cancer. Lung Cancer. 2019;132:65‐71. 31097096 10.1016/j.lungcan.2019.04.005

[37] Facchinetti F , Bluthgen MV , Tergemina‐Clain G , et al. LKB1/STK11 mutations in non‐small cell lung cancer patients: Descriptive analysis and prognostic value. Lung Cancer. 2017;112:62‐68. 29191602 10.1016/j.lungcan.2017.08.002

[38] Papillon‐Cavanagh S , Doshi P , Dobrin R , Szustakowski J , Walsh AM . STK11 and KEAP1 mutations as prognostic biomarkers in an observational real‐world lung adenocarcinoma cohort. ESMO Open. 2020;5:e000706. 32312757 10.1136/esmoopen-2020-000706 PMC7199918

[39] Skoulidis F , Goldberg ME , Greenawalt DM , et al. STK11/LKB1 mutations and PD‐1 inhibitor resistance in KRAS‐mutant lung adenocarcinoma. Cancer Discov. 2018;8:822‐835. 29773717 10.1158/2159-8290.CD-18-0099 PMC6030433

[40] Biton J , Mansuet‐Lupo A , Pécuchet N , et al. TP53, STK11, and EGFR mutations predict tumor immune profile and the response to Anti‐PD‐1 in lung adenocarcinoma. Clin Cancer Res. 2018;15(24):5710‐5723. 10.1158/1078-0432.CCR-18-0163 29764856

[41] Lee CK , Man J , Lord S , et al. Clinical and molecular characteristics associated with survival among patients treated with checkpoint inhibitors for advanced non‐small cell lung carcinoma: a systematic review and meta‐analysis. JAMA Oncol. 2018;1(4):210‐216. 10.1001/jamaoncol.2017.4427 PMC5838598 29270615

[42] Friedrich M.J. . Going With the Flow: The Promise and Challenge of Liquid Biopsies. JAMA. 2017;318 (12):1095. 28885642 10.1001/jama.2017.10203

[43] Cabel L , Riva F , Servois V , et al. Circulating tumor DNA changes for early monitoring of anti‐PD1 immunotherapy: a proof‐of‐concept study. Ann Oncol. 2017;1(28):1996‐2001. 10.1093/annonc/mdx212 28459943

[44] Goldberg SB , Narayan A , Kole AJ , et al. Early assessment of lung cancer immunotherapy response via circulating tumor DNA. Clin Cancer Res. 2018;15(24):1872‐1880. 10.1158/1078-0432.CCR-17-1341 PMC5899677 29330207

[45] Raja R , Kuziora M , Brohawn PZ , et al. Early reduction in ctDNA predicts survival in patients with lung and bladder cancer treated with durvalumab. Clin Cancer Res. 2018;15(24):6212‐6222. 10.1158/1078-0432.CCR-18-0386 30093454

[46] Giroux Leprieur E , Herbretau G , Dumenil C , et al. Circulating tumor DNA evaluated by next‐generation sequencing is predictive of tumor response and prolonged clinical benefit with nivolumab in advanced non‐small cell lung cancer. Oncoimmunology. 2018;7:e1424675. 29721388 10.1080/2162402X.2018.1424675 PMC5927532

[47] Wang Z , Duan J , Cai S , et al. Assessment of blood tumor mutational burden as a potential biomarker for immunotherapy in patients with non‐small cell lung cancer with use of a next‐generation sequencing cancer gene panel. JAMA Oncol. 2019;1(5):696‐702. 10.1001/jamaoncol.2018.7098 PMC6512308 30816954

[48] Gandara DR , Paul SM , Kowanetz M , et al. Blood‐based tumor mutational burden as a predictor of clinical benefit in non‐small‐cell lung cancer patients treated with atezolizumab. Nat Med. 2018;24:1441‐1448. 30082870 10.1038/s41591-018-0134-3

[49] Nicolazzo C , Raimondi C , Mancini M , et al. Monitoring PD‐L1 positive circulating tumor cells in non‐small cell lung cancer patients treated with the PD‐1 inhibitor nivolumab. Sci Rep. 2016;6:31726. 27553175 10.1038/srep31726 PMC4995431

[50] Guibert N , Delaunay M , Lusque A , et al. PD‐L1 expression in circulating tumor cells of advanced non‐small cell lung cancer patients treated with nivolumab. Lung Cancer. 2018;120:108‐112. 29748004 10.1016/j.lungcan.2018.04.001

[51] Hofman P , Heeke S , Alix‐Panabières C , Pantel K . Liquid biopsy in the era of immuno‐oncology: is it ready for prime‐time use for cancer patients? Ann Oncol. 2019;1(30):1448‐1459. 10.1093/annonc/mdz196 31228184

[52] Kargl J , Busch SE , Yang GH , et al. Neutrophils dominate the immune cell composition in non‐small cell lung cancer. Nat Commun. 2017;1(8):14381. 10.1038/ncomms14381 PMC5296654 28146145

[53] Akbay EA , Koyama S , Liu Y , et al. Interleukin‐17A promotes lung tumor progression through neutrophil attraction to tumor sites and mediating resistance to PD‐1 blockade. J Thorac Oncol. 2017;12:1268‐1279. 28483607 10.1016/j.jtho.2017.04.017 PMC5532066

[54] Bagley SJ , Kothari S , Aggarwal C , et al. Pretreatment neutrophil‐to‐lymphocyte ratio as a marker of outcomes in nivolumab‐treated patients with advanced non‐small‐cell lung cancer. Lung Cancer. 2017;106:1‐7. 28285682 10.1016/j.lungcan.2017.01.013

[55] Diem S , Schmid S , Krapf M , et al. Neutrophil‐to‐Lymphocyte ratio (NLR) and Platelet‐to‐Lymphocyte ratio (PLR) as prognostic markers in patients with non‐small cell lung cancer (NSCLC) treated with nivolumab. Lung Cancer. 2017;111:176‐181. 28838390 10.1016/j.lungcan.2017.07.024

[56] Kamphorst AO , Pillai RN , Yang S , et al. Proliferation of PD‐1+ CD8 T cells in peripheral blood after PD‐1‐targeted therapy in lung cancer patients. Proc Natl Acad Sci USA. 2017;9(114):4993‐4998. 10.1073/pnas.1705327114 PMC5441721 28446615

[57] Huang AC , Postow MA , Orlowski RJ , et al. T‐cell invigoration to tumour burden ratio associated with anti‐PD‐1 response. Nature. 2017;4(545):60‐65. 10.1038/nature22079 PMC5554367 28397821

[58] Kagamu H , Kitano S , Yamaguchi O , et al. CD4(+) T‐cell immunity in the peripheral blood correlates with response to Anti‐PD‐1 therapy. Cancer Immunol Res. 2020;8:334‐344. 31871122 10.1158/2326-6066.CIR-19-0574

[59] Zappasodi R , Budhu S , Hellmann MD , et al. Non‐conventional Inhibitory CD4(+)Foxp3(‐)PD‐1(hi) T cells as a biomarker of immune checkpoint blockade activity. Cancer Cell. 2018;11(33):1017‐1032.e7. 10.1016/j.ccell.2018.05.009 PMC6648657 29894689

[60] Thommen DS , Koelzer VH , Herzig P , et al. A transcriptionally and functionally distinct PD‐1+ CD8+ T cell pool with predictive potential in non‐small‐cell lung cancer treated with PD‐1 blockade. Nat Med. 2018;24:994‐1004. 29892065 10.1038/s41591-018-0057-z PMC6110381

[61] Fehlings M , Jhunjhunwala S , Kowanetz M , et al. Late‐differentiated effector neoantigen‐specific CD8+ T cells are enriched in peripheral blood of non‐small cell lung carcinoma patients responding to atezolizumab treatment. J Immunother Cancer. 2019;12(7):249. 10.1186/s40425-019-0695-9 PMC6740011 31511069

[62] Costantini A , Julie C , Dumenil C , et al. Predictive role of plasmatic biomarkers in advanced non‐small cell lung cancer treated by nivolumab. Oncoimmunology. 2018;7:e1452581. 30221046 10.1080/2162402X.2018.1452581 PMC6136870

[63] Costantini A , Takam Kamga P , Dumenil C , Chinet T , Emile JF , Giroux LE . Plasma biomarkers and immune checkpoint inhibitors in non‐small cell lung cancer: new tools for better patient selection? Cancers. 2019;29:11. 10.3390/cancers11091269 PMC6769436 31470546

[64] Kim DH , Kim H , Choi YJ , et al. Exosomal PD‐L1 promotes tumor growth through immune escape in non‐small cell lung cancer. Exp Mol Med. 2019;51:1‐13. 10.1038/s12276-019-0295-2 PMC6802663 31399559

[65] Yu S , Shi M , Feng J . 138P ‐ Expression of PD‐L1 in plasma exosomes of NSCLC patients and its associations with PD‐L1 expression of corresponding tumour tissues. Ann Oncol. 2019;30:v43.

[66] Gunasekaran M , Russo A , Cardona AF , et al. Exosomal PD‐L1 expression as non‐invasive biomarker for immune checkpoint inhibitors in non‐small cell lung cancer. J Immunol. 2020;204:90.10.

[67] Sanmamed MF , Perez‐Gracia JL , Schalper KA , et al. Changes in serum interleukin‐8 (IL‐8) levels reflect and predict response to anti‐PD‐1 treatment in melanoma and non‐small‐cell lung cancer patients. Ann Oncol. 2017;1(28):1988‐1995. 10.1093/annonc/mdx190 PMC5834104 28595336

[68] Chen H , Chong W , Teng C , Yao Y , Wang X , Li X . The immune response‐related mutational signatures and driver genes in non‐small‐cell lung cancer. Cancer Sci. 2019;110:2348‐2356. 31222843 10.1111/cas.14113 PMC6676111

[69] Richard C , Fumet JD , Chevrier S , et al. Exome analysis reveals genomic markers associated with better efficacy of nivolumab in lung cancer patients. Clin Cancer Res. 2019;1(25):957‐966. 10.1158/1078-0432.CCR-18-1940 30154227

[70] Higgs BW , Morehouse CA , Streicher K , et al. Interferon gamma messenger RNA signature in tumor biopsies predicts outcomes in patients with non‐small cell lung carcinoma or urothelial cancer treated with durvalumab. Clin Cancer Res. 2018;15(24):3857‐3866. 10.1158/1078-0432.CCR-17-3451 29716923

[71] Prat A , Navarro A , Paré L , et al. Immune‐related gene expression profiling after PD‐1 blockade in non‐small cell lung carcinoma, head and neck squamous cell carcinoma, and melanoma. Can Res. 2017;1(77):3540‐3550. 10.1158/0008-5472.CAN-16-3556 28487385

[72] Karachaliou N , Gonzalez‐Cao M , Crespo G , et al. Interferon gamma, an important marker of response to immune checkpoint blockade in non‐small cell lung cancer and melanoma patients. Ther Adv Med Oncol. 2018;10:1758834017749748. 29383037 10.1177/1758834017749748 PMC5784541

[73] Wang S , Jia M , He Z , Liu XS . APOBEC3B and APOBEC mutational signature as potential predictive markers for immunotherapy response in non‐small cell lung cancer. Oncogene. 2018;37:3924‐3936. 29695832 10.1038/s41388-018-0245-9 PMC6053356

[74] Ayers M , Lunceford J , Nebozhyn M , et al. IFN‐γ‐related mRNA profile predicts clinical response to PD‐1 blockade. J Clin Investig. 2017;1(127):2930‐2940. 10.1172/JCI91190 PMC5531419 28650338

[75] Hwang S , Kwon AY , Jeong JY , et al. Immune gene signatures for predicting durable clinical benefit of anti‐PD‐1 immunotherapy in patients with non‐small cell lung cancer. Sci Rep. 2020;20(10):643. 10.1038/s41598-019-57218-9 PMC6971301 31959763

